# The Clip Approach: A Visual Methodology to Support the (Re)Construction of Life Narratives

**DOI:** 10.1177/1049732320982945

**Published:** 2021-02-11

**Authors:** Riikka Talsi, Aarno Laitila, Timo Joensuu, Esa Saarinen

**Affiliations:** 1 Aalto University, Espoo, Finland; 2 University of Jyväskylä, Jyväskylä, Finland; 3 Docrates Cancer Center, Helsinki, Finland

**Keywords:** life narrative, illness experience, autobiographical rupture, autobiographical reasoning, narrative identity, interview methodology, visual artifact, interventive interview, prostate cancer, spouse, psychosocial support method, qualitative, narrative-hermeneutic method, Finland

## Abstract

Major life changes may cause an autobiographical rupture and a need to work on one’s narrative identity. This article introduces a new qualitative interview methodology originally developed to facilitate 10 prostate cancer patients and five spouses in the (re)creation of their life narratives in the context of a series of interventive interviews conducted over a timespan of several months. In “The Clip Approach” the interviewees’ words, phrases, and metaphors are reflected back in a physical form (“the Clips”) as visual artifacts that allow the interviewees to re-enter and re-consider their experience and life and re-construct their narratives concerning them. Honoring the interviewees as authors facilitates autobiographical reasoning, building a bridge between the past and the future, and embedding the illness experience as part of one’s life narrative. The Clip Approach provides new tools for both research and practice—potentially even a low-threshold psychosocial support method for various applicability areas.

## Introduction

Narratives and life stories are created individually and in interaction, they form a narrative identity, which develops slowly over time and gives a feeling of wholeness and meaning to life (McAdams, 2013; McAdams & McLean, 2013; Sparkes & Smith, 2008). Getting seriously ill, and surviving or recovering from a serious illness, is an autobiographical rupture (Bury, 1982), which a human being seeks to correct by building a link between the past and the future. In conventional life, the life story is nonconscious. One begins to ponder it consciously, when it becomes distorted (Hänninen, 2004). Then also telling the story becomes topical (Frank, 1995).

Mere remembering of past episodes is insufficient (Habermas & Köber, 2015), one needs to interpret and evaluate remembered experiences (Singer & Bluck, 2001) and make connections between distant parts of life, self, and personal development (Habermas & Köber, 2015; McLean & Fournier, 2008). Autobiographical reasoning facilitates coping and contributes to the development of personality, identity (Habermas, 2011), and personal growth (McLean & Fournier, 2008). Life stories showing agency, exploration, and redemptive meanings seem to relate to higher levels of mental health, well-being, and maturity (Kerr et al., 2020; McAdams & McLean, 2013). Even without a positive resolution, negative experiences may contribute to well-being by enhancing meaning through stimulated comprehension of how the event fits into a broader narrative of self, relationships, and the world (Vohs et al., 2019). Narrative identity is at the core of adaptation and development (McAdams & McLean, 2013), and storytelling is at the heart of stability and change in the self (McLean et al., 2007).

All counseling and psychotherapy rely on telling stories and, in the end, re-telling the client’s life story (McLeod, 1996) as a collaborative task (Kohler Riessman & Speedy, 2006). Narrative therapy practice is a form of “live” co-research, described by others as narrative inquiry (Kohler Riessman & Speedy, 2006). It studies the human process of meaning-making and is especially suitable for inquiring into these processes regarding the self (Adler et al., 2017). In qualitative inquiry, subjects are treated as and called participants, and the research process itself is viewed as a type of intervention (Gale, 1993). Qualitative inquiry may include some, possibly several change processes that have been described as helpful by clients in therapy (Timulak, 2010). As the pluralistic framework of practice, it makes use of the therapeutic possibilities existing in the wider culture in which people live—using what the participant knows, taking it seriously (McLeod, 2013).

### Visual Artifacts

Qualitative inquiry potentially benefits from the visual material used, created, or produced in collaboration. There is a growing interest in using visual methodologies and creative artifacts in social research; examples range from photography, documentary filmmaking, paintings, graffiti, and computer-mediated interactions (Reavey & Johnson, 2011) to Lego constructions (Gauntlett & Holzwarth, 2006). In narrative research, visual timelines have been used to locate main events during a specified timeframe (Black & Warhurst, 2015). Narrative psychotherapy in turn utilizes, for example, certificates and declarations as therapeutic documentation (Morgan, 2000).

In psychological research, visual methodologies are a salutary way of accessing meaning and expressing and transforming experience (Reavey & Johnson, 2011). They provide new ways of experiencing and creative mediums to generate meanings; instead of only speaking, participants are able to see their world in a tangible dimension, offering a channel for engaging the experience from the nondiscursive side of producing oneself (Reavey & Johnson, 2011). Visual artifacts are symbolic representations through which ideas can be articulated, developed, and exchanged; they provide material entities with which to interact and develop knowledge (Ewenstein & Whyte, 2007). Visual approaches strengthen participant-agency (Reavey & Johnson, 2011) and invite the practice of self-reflection more freely (Mitchell et al., 2005). They may also develop feelings of artistry, creativity, and productivity (Predeger, 1996).

### Interview as an Interaction

Serial interviews allow the participant–researcher relationship to develop and deepen over time, creating trust and enabling participants to voice sensitive issues in their personal accounts (Murray et al., 2009). In qualitative interviews, participants have an engaged audience, they get listened to. Qualitative inquiry appreciates (Adler et al., 2017), even amplifies participants’ voices (Martin, 1998). Emphasizing the absolute authority of the narrator as to how to build the story, Martin (1998) aptly writes: “To speak is one thing, to be heard is another, to be confirmed as being heard is yet another” (p. 9). Telling one’s story may be an empowering act (Martin, 1998), and acceptance shown in an interview carries healing potential (Tomm, 1988).

Serial interviews support self-reflection by allowing earlier findings to be developed and reflected on (Murray et al., 2009). A necessary step toward being able to act upon a phenomenon is that of first becoming an observer through which both self-awareness and “other” awareness, like empathy, is enhanced (Tomm, 1987b). In Tomm’s (1987a) concept of “interventive interviewing” regarding the entire interview as a series of continuous interventions, reflexive questions are central (Tomm, 1988). Respecting the autonomy of the participants, they open space for new perceptions, perspectives, and options, creating new connections and fresh solutions, and guiding the participants toward releasing their innate healing capacity in their own manner and time (Tomm, 1988). Serial interviews allow narratives to unfold forming rich, generative, and contextualized accounts (Murray et al., 2009) with “a nearly endless opportunity for revisiting and for discovery” (Adler et al., 2017, p. 524). It is important that rather than writing the last chapter of the story, the research reports allow the stories to continue (Sparkes & Smith, 2008).

## Research Task

This article introduces a new qualitative interview methodology originally developed to facilitate prostate cancer patients and their spouses in the (re)creation of their life narratives in the context of a series of interventive interviews. The focus is on the use of visual artifacts (“the Clips”) introduced and developed by Riikka Talsi to reflect the richness of the interviewees’ accounts and to help the interviewees to re-enter and re-evaluate their experience and re-construct their life story. The aim of the present article is to demonstrate the usefulness of the Clips in an interview process designed to support people in their efforts to make sense of their experience and to come to terms with potentially traumatic life events. The philosophical base of the study is social constructionist and interpretivist. The methodological base is narrative and hermeneutic.

## Method

### Participants

Seeing severe illness as a family matter, a dyadic concern (Persson et al., 2020), an option to participate as a couple was provided. Ten newly diagnosed prostate cancer patients were recruited to participate in narrative in-depth interviews, five of them individually, and five with their spouse. All new prostate cancer patients filling the inclusion criteria of a maximum 3 months from the diagnosis and a curative treatment as a target were systematically informed of the research during the first medical appointment with Timo Joensuu. In addition, a study announcement was published on a hospital website and in patient organizations. Interested patients and spouses were given a study fact sheet, and they signed an informed consent form.

To ensure participant anonymity, demographic data are presented as group data (Morse, 2007). All patients were treated with external radiotherapy, two of them also twice with internal high dose rate (HDR) brachytherapy. The external radiotherapy lasted for 8 weeks with treatment being given every weekday; during brachytherapy, external radiation was suspended. One patient was treated with docetaxel chemotherapy before external radiotherapy. All patients except one were also treated with hormonal therapy. The time from the diagnosis ranged from 1 week to 2 months. The ages of the patients ranged from 48 to 74 years (mean 61.9) and the ages of the spouses from 48 to 70 years (mean 61.4). The duration of the marriages of the couples involved ranged from 2 to 50 years. One of the patients was divorced, and two patients went through a divorce process during the period of data collection. The number of children in common ranged from none to three, and from previous marriages from one to four per couple. Both patients and spouses came from various occupational backgrounds a minority of them having an academic degree. Four patients and two spouses were retired. One patient also sought help from a peer support group.

### Data Collection

Each patient/couple participated in five interviews during the cancer treatment period, and a follow-up interview a year after the first interview. The five narrative in-depth interviews were scheduled either tightly over 2 months’ radiotherapy or during a half year period (for one participant 4 months) including both radiotherapy and partly hormone therapy (for one participant also chemotherapy). The two main options enabled assessing as to whether frequent interviews or a schedule giving more time for reflection between the meetings, served the interviewees better. Furthermore, frequent interviews during radiotherapy minimized the travel requirements of those participants from further away, yet some of them selected a longer interview period. In all, the interview periods were split, evenly, between the two alternatives. Interview intervals within an agreed interview period were suited to the interviewees’ needs. The interviews were carried out in Finnish.

The interviews were designed and conducted by Riikka Talsi with the design being confirmed by Aarno Laitila. Riikka Talsi is a doctoral candidate with a long professional career in training, coaching, and leading people together with a recent 5-year experience in research and development. She has studied science, psychology, and medicine. In terms of the cancer treatment, Riikka Talsi was a complementary person for whom research permission had been granted to find new holistic directions in cancer treatment.

In the interviews, I (Riikka Talsi) asked the interviewees to reflect on their lives, the cancer experience, its effects and the needs arising from it, freely, according to and emphasizing what they felt to be of significance. The interview could start with a simple life story prompt: “Could you tell me your life story?” (used once with all the interviewees) or be initiated using the following main themes: cancer treatment; physical, mental, and social well-being; family, patient, and spouse; work and leisure time; the future; cancer experience; and the life story from different viewpoints. I raised the themes according to the interviewees’ present concerns at the time of the interview, however, making sure that all main themes were addressed during the interview period. When necessary, I continued by asking clarifying questions or presented subthemes as narrative topics (see Supplemental Attachment 1).

The interview methodology includes three context-situated ideas not reported in earlier studies. The first one arose in the first pilot interview, when I asked the spouse by which metaphor would she describe the cancer experience within the whole of her life. The metaphor she gave, and the dialogue following, was so inspiring that I decided to include the “Momentary key metaphor” in all the interviews. These are assessed here to the extent needed to report the new methodology. I finished each interview by asking, with which metaphor would the interviewee describe the cancer experience at that moment in his or her life. This was done by continuing the following sentence: “Being ill with cancer in my life is like . . . ” (patient)/“My spouse being ill with cancer, in my life, is like . . . ” (spouse). Then, I asked the interviewee to illustrate the meaning of the metaphor. In many cases, the metaphors directly presented two contrasting modes, for example “*white puffy clouds, blue sky behind—there is light, behind the clouds there is light*” (clouds – light), or could be understood as such, for example “*trying to wake up from a bad dream*” (being in the midst of a nightmare – being awake). They seemed to harbor the current and the desired. This perception led me to the next idea of developing “Dual lighting”—interviewing through a metaphorical window. In this method, I asked the interviewees to contemplate their life and the cancer experience through the introduction of the aforementioned interview themes through their earlier given metaphor. I could for example ask: “In what areas of life and this experience, do you already see light, and in what areas are there still clouds?” and then we would go through the interview themes. Dual lighting was used as a window to life aiming at supporting the interviewees in seeing their progress in the process.

An unconditional respectfulness for the interviewees, their lives, experiences and what they brought forth, as well as careful, responsive listening, were important principles for me throughout the interviews. I listened, personally transcribed, and summarized each interview before the next one took place, so that I could recall important excerpts and thoughts from the previous interview, and thus support the interviewee(s) in building a broader picture. I conducted the interviews in a reflective mode, and when possible, summarized the content of the ongoing interview to the interviewee(s). The interviewees were given as much time as they felt they needed, the duration of the interviews ranging from 42 to 160 minutes (mean 95 min).

When preparing for the first time for the fifth interview, I went through all the preceding interviews of that specific couple, immersing myself in the data in the same empathizing way I had listened to them in the interviews. All the phases, the events, and happenings in their lives, and their way of telling it in their own personal voices. The challenge was of how to reflect it all, of how to help and support them in taking these fundamental building blocks and in re-constructing their own story. Then I came up with the third idea. Just as I had orally recalled important excerpts and thoughts from the previous interviews and brought them to the next one, I would pick out salient words, metaphors, and sentences from all their previous interviews and write them down on slips of paper. Then, I would set the Clips on a table in a random order and ask them to build their life story including the cancer experience, as they perceived it at that specific moment, using the excerpts they felt meaningful for the story, leaving others unused. I would also make a pile of empty slips so that they could add new words and sentences to their story, telling them that the form of the story is completely free and that they could construct a common story or two individual ones, just as they wished.

Selecting the Clips took me to the responsive listening mode on a higher level. At that point, I had already met the interviewees four times, become acquainted with them as people and come to understand their individual ways of being, behaving, speaking, and responding. Their life stories and the cancer experience had emerged and been discussed in a variety of ways. In preparing the Clips, I went through the interview transcriptions, with the larger context informed by hermeneutics in mind, underlining potential Clip candidates: interviewees’ personal ways of expressing life and its events; self and others; the past and the future; feelings and thoughts; options and choices; evaluations and perceptions; questions and answers; values, goals, and wishes; meanings and metaphors. The first round resulted in more pieces of text than I thought would be a manageable number of Clips. I went through the underlined texts iteratively several times, pruning any that overlapped and the less central ones, with the objective of identifying the most salient quotations presenting the accounts as the result. I had to make choices, but I made them with my best understanding with the aim of doing justice to who the interviewees were as people, what they had experienced and how they had told what they had told. The Clips were my choices, which I brought for the interviewees’ evaluation, whether sufficient and striking the right chord, remained to be assessed by the interviewees.

I was encouraged by the simple impressive stories the couple, previously referred to, created using the Clips. The man quickly produced his story, which to my surprise followed the structure of a story defined by Labov (2006), although he had probably never heard of it. For the spouse, it took a little longer to get started, but she, too, was able to create an impressive personal narrative. The unique stories expressed causal connections, re-used personal narratives to recognize and strengthen one’s agency, evaluated experience, and interpreted meaning. Heartened by the way both could re-utilize their rich telling beyond what the pieces of narratives were as independent parts, I decided to use the method with all the interviewees.

The follow-up interviews examined the cancer experience retrospectively, but also brought forth new dimensions and considerations. First, we discussed the progress of the interviewees’ well-being and quality of life during the follow-up period. Then I asked the participant(s) for a Momentary key metaphor, with the prompt being used in the past tense. After that I handed the participant(s) a paper with his or her personal Momentary key metaphors in chronological order and asked him or her to reflect on the metaphors and to ponder whether they created a story line. Thereafter, we went back to the story constructed with the Clips in the fifth interview (hereafter “the Storyboard”), which I had rebuilt on a table according to a photograph taken at the time, and I asked the interviewee(s) to review the story, change it if needed, and potentially continue it. We then discussed the meaning of the interviews in processing the cancer experience and life, and the potential posttraumatic growth created by the cancer experience. Finally, I checked whether there was something the interviewee(s) would still like to add.

I made research journal entries immediately after each interview, which provided me with important moments to pause, to reflect, and to process how I had felt upon the interviews and what kinds of thoughts and emotions had arisen in me. I returned to the entries between the interviews, when preparing the Clips, and also during the data analysis. The entries turned out to be beneficial in various ways: I could check some details through them, they supported me in responsive listening, and they helped me in recognizing, distinguishing, and being aware of my own feelings and attitudes. I carried out the main interviews during February–August 2015 and the follow-up interviews during February–July 2016. The data consist of 60 audio-recorded and transcribed interviews (100 hours), 12 Storyboards, and 95 Momentary key metaphors.

### Validity

The trustworthiness and authenticity of the study was built on several validity procedures presented for qualitative inquiry in literature (Creswell & Miller, 2000): (a) close collaboration with the participants throughout the research process with the intent to respect and support them; (b) prolonged engagement in the field building a tight and holistic case; (c) researcher reflexivity; (d) member checking—participants validated and commented on the accuracy of the Clips as raw data and the Storyboards as accounts; (e) triangulation from several perspectives—from multiple data sources (participants) and through multiple methods (observations, interviews, and documents—with the Storyboards and Clips as textual and visual artifacts); (f) thick and rich description of the setting, the participants, the development of the new methodology, the interview process, and the data. The article presents several Clips, excerpts, and photographs of the Storyboards, as well as interview excerpts to enable the evaluation of the results and interpretations and conclusions drawn from them. Furthermore, the role of the researcher, in developing the methodology, in the interviews, and in the construction of the stories has been pointed out and illustrated.

### Ethical Approval

The research was approved by the Ethics Committee for gynecology and obstetrics, pediatrics, and psychiatry of the Hospital District of Helsinki and Uusimaa (161/13/03/03/2014).

## Analysis

The analysis was performed using both data and theory-driven procedures. It was carried out with a continuous dialogue between the data, and the narrative theory and change process frameworks presented in the introduction. The results were collided from different perspectives—like particles collided in particle physics to produce new ones—then examined using targeted counter-argumentation, and finally refined in repeated team discussions. The aim of this article, however, is not to present a detailed analysis of the (re)created stories, instead based on observational data, participant feedback, as well as story excerpts, to present upper-level constructs emerging in the data when the participants of this study used the Clip Approach.

## Results

The interview methodology offers several tools to re-enter and re-consider one’s experience and life and to re-construct one’s narratives concerning them. The results section focuses on how building one’s story with the Clips supported the interviewees in these human endeavors. We first set the scene and present the various ways in which the interviewees applied the Clip Approach. Then, we illustrate, how the Clip Approach as a visual method facilitated the participants in returning to their narrative, in exploring and re-evaluating meaning, and in re-constructing an evolving life story embedding the cancer experience.

### The Individual Ways of Applying the Clip Approach in the Sample

The number of Clips ranged from 60 to 102 per patient or couple. As couples could construct one common Storyboard or two individual ones, at some point of the process, they needed to decide as to which would best serve their purpose. Two couples decided to build a shared story including Clips from both spouses. In two other cases, the husband started building his individual story; the spouse noticed this and started to create hers. In one of these cases, however, the stories included Clips from both spouses. The fifth couple first built two individual stories, which the wife later united.

Constructing the story in the fifth interview took place by selecting and organizing Clips. On average, more than 4/5 (607/803~83.4%) of the Clips were used, six interviewees (two couples) using all their Clips. Two interviewees added new Clips to their stories, and two interviewees inserted new text onto original Clips. Five Clips fitting two places in the story of one participant were duplicated. One couple removed two words from one of their Clips.

**Figure 1.**
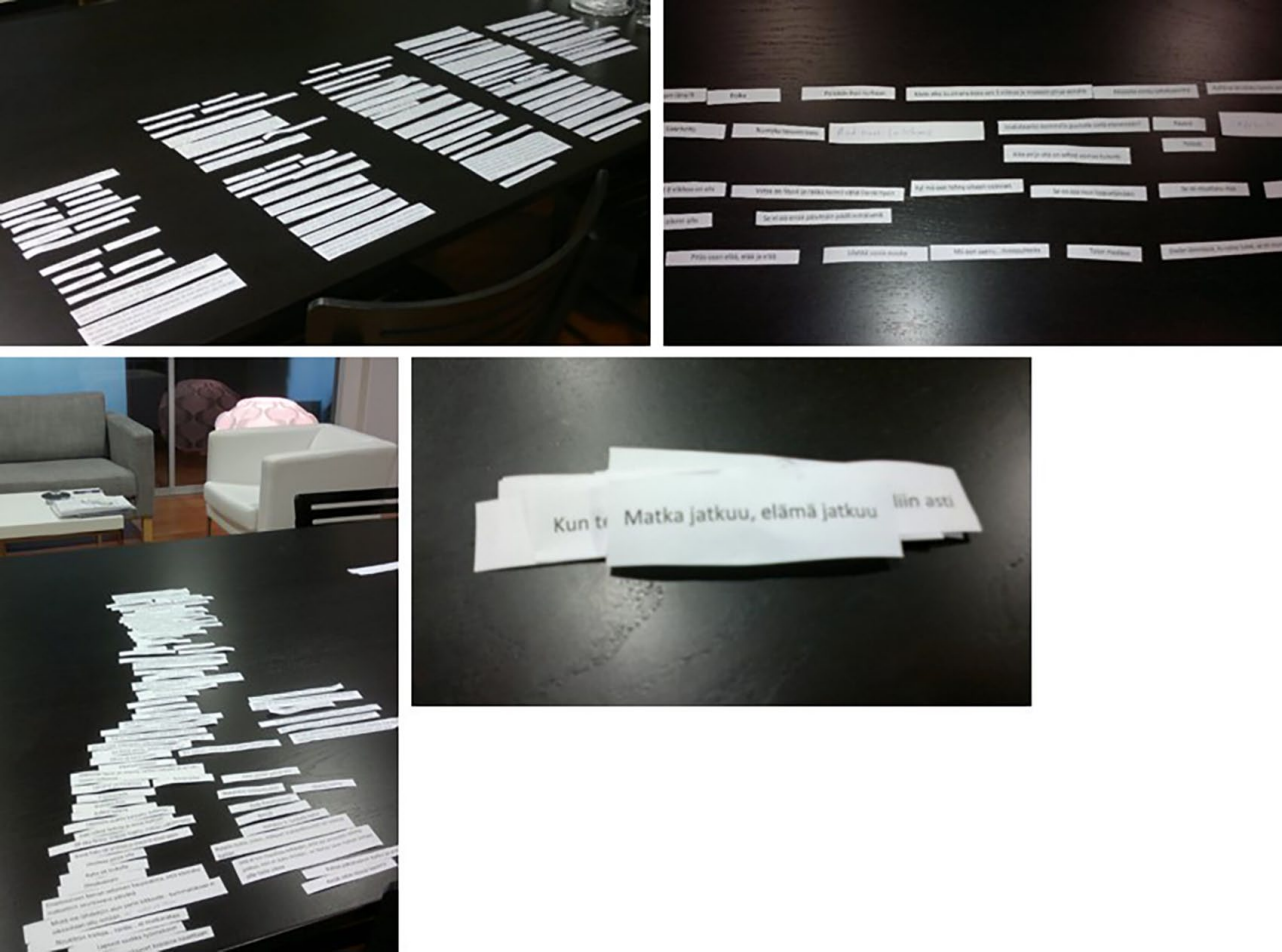
Stories in different shapes: in columns, in rows, in an unclassified form, and as a pile.

Participants organized the Clips on the provided surface in several ways leading to stories in a variety of visual forms: in columns, in rows, in a pillar, in clusters, in an unclassified form, even in a pile. The reading direction could go downward or upward, but horizontally it went always to the right. There could be parallel Clips inside the columns or a pillar and above or under the rows—even perpendicular to other Clips. There could also be open space and vertical paralleling inside a story. Figure 1 presents examples of the visual variety of the stories.

All the stories were continued in the follow-up interview, five of them vocally with no changes to the Storyboard. One story was continued adding a new Clip in the middle of the story and three others adding new Clips at the end of it. Other stories were continued using Clips from the original story, all in different ways: by organizing several existing Clips with no textual changes in a new order at the end of the story; by removing text from and adding new text to one Clip and moving it to the end of the story; and by composing a continuation from several new Clips and original ones with complementary text being added to them. In addition, one participant changed the chronological order of four Clips in his personal history.

### Returning to One’s Narrative in a Visual Form

. . . these [the Clips] . . . bring exactly into mind what we have storied here (patient, the fifth interview)

The interviewees were amazed when they encountered the Clips for the first time in the fifth interview. They recognized the Clips as their own at once and remembered the stories behind them, finding the Clips as meaningful and authentic reflections of one’s own thinking:

. . . quite essential points have been found there . . . when one remembers what one has said, then they have been somehow important (intonation going up, breathing in) . . . there is no bluff . . . here is exactly what we have thought (spouse, the fifth interview)

The work-intensive background work, which culminated in the Clips in the fifth interview, provided the participants with “feedforward” helping them to make sense of their experience:

. . . the fact that you have, in the background, made these summaries and put effort into writing the comments [the Clips] on the paper, and put them here, it is that kind of feedback, which is totally unique, it helps seeing it much better . . . if I would now talk with a psychotherapist . . . we would not go through comments that I have said a year ago . . . because we wouldn’t have these in the same way . . . it is not at all the same as this type of a summary and feedback (patient, the follow-up interview)

The Clips transformed the oral accounts into a tangible, visually approachable and movable form, inviting fresh reflection and dialogue. Picking out a Clip and starting to ponder on its significance and position with respect to other Clips strengthened the interviewees’ agency. Participants recognized that the physical organizing that the Clips called for was intertwined with mental work through which one’s narrative and life itself could be organized in new ways:

. . . it brings that kind of a concreteness to one’s own affairs . . . that someone puts so much effort in going through my texts . . . and prints them out on a paper and I get to sort them . . . yes, yeah, it has helped in structuring (patient, the fifth interview)

On one hand, the Clips created a beneficial observer perspective, whereas on the other they enabled re-entering one’s feelings, bringing forth affection and appreciation for one’s journey:

One thinks from a bit longer distance, sees even oneself, what has said, and realizes how it has been of a **benefit** (emphasizing), it really has been of a benefit (spouse, the fifth interview)

. . . I think, that exactly, when they [the Clips] are our own sentences and thoughts and feelings and so forth, so I think it was terribly good, and like, oh, yes, it went like this, and somehow one is again maybe able to somehow (gets moved, starts crying) appreciate this journey more (spouse, the fifth interview)

The Clips offered a means of re-entering one’s experience also in the future: “*Will you give these pieces to take with me?*” asked one participant at the end of his fifth interview and took a photo of his Storyboard. Furthermore, seeing one’s speech as a text made some participants think that they should write a book, the story as depicted by the Clips being a good start for it.

### Construing One’s Life History and the Wider Frame of Life

This is not just about cancer for me,

it is about the structures of my entire life and how

I can respond to this tough struggle.

(a Clip of a story of a patient, the fifth interview)

The Clip Approach enabled construing one’s life history and the formation of a wider frame of the present life. Most Storyboards included autobiographical reasoning on life history — such as childhood and youth, relationships, family life, work, and previous challenges in life—but also current issues outside of cancer, such as searching for oneself, alcoholism, or a divorce. Besides cancer, participants could be struggling with other life challenges shaking their identity and calling for a scrutiny on fundamental life themes like targets, the way of living, interdependency, and the self. The Clips supported exploring and considering life, relationships and self, and making and expressing conclusions (patient, the follow-up interview, hand-written text in Ink Free font):


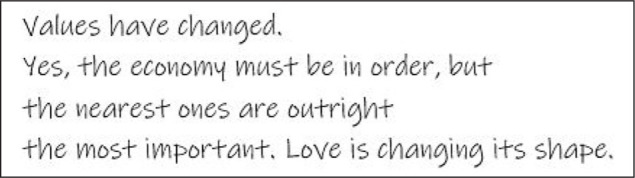


### Structuring the Cancer Experience

Is this the end?

(a Clip of a story of a couple, the fifth interview)

A recurring phenomenon in the material was the handling of the disruption caused by the illness to one’s life story. All the Storyboards discussed the cancer experience and feelings related to it, from the moment of hearing the diagnosis with fears of death (patients) and being left alone (spouses), to seeking for a treatment through sometimes difficult consideration between treatment options, then illustrating the treatment period, together with empowering things, and at some point in the story recognizing agency and confidence in oneself, finally entering recovery with feelings of relief, trust, gratitude, and hopefulness. An illustration of how the interviewees structured the cancer experience is presented in Supplemental Attachment 2 as a composition of short extracts of several Storyboards of several participants, all from the fifth interviews.

### Exploring and Re-Evaluating Meaning

Something gets a totally new meaning now.

Possibly, if I will recover, an even bigger meaning

(a Clip of a story of a patient, the fifth interview)

With the Clips, the interviewees could explore and re-evaluate the illness and come to new interpretations for it. “*The word cancer is no more equivalent to death to me*” as one participant expressed it on a Clip. The Clips enabled the expression of strength, perseverance, and survival in oneself allowing for new interpretations for the illness experience (couple, the fifth interview):

All those difficult situations in this kind of a crisis, turn into a victory when one realizes that, for heaven’s sake, I have grown there and developed and changed and gained maturity and depth

**Figure 2.**
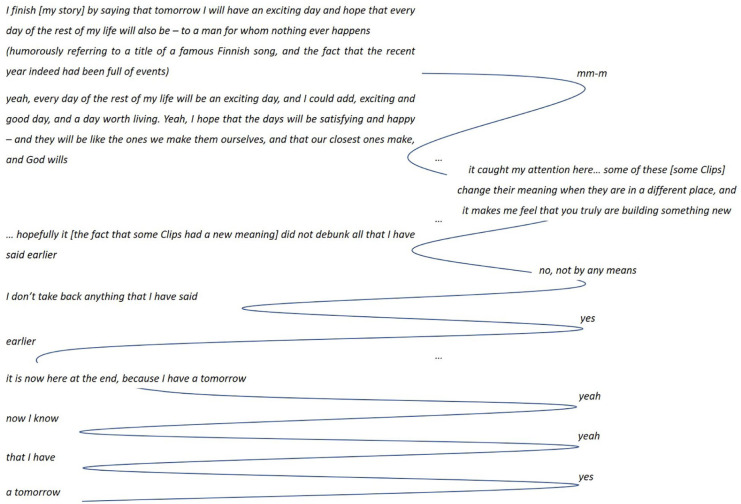
Interview extract from the fifth interview, participant cited on the left, interviewer cited on the right.

The cancer experience recurrently got a meaning related to something new—“*Searching for something new*,” finding or building new, understanding in a new way, even “*Being born again*”—and the Clips offered a chance to connect it to this new perspective. This transformative aspect of the Clips is illustrated in the above interview excerpt at which the very meaning of a Clip evolves during the process. “*Tomorrow I will have an exciting day*” stated in the distressed situation of the day before hearing the results of the magnetic resonance imaging in a suspect of a wide metastasizing, turned out to represent possibilities and the future (Figure 2).

The dialogue builds on the process the interviewee had gone through during the previous months—a long marriage slowly going on the rocks against his own will, cancer diagnosis and the treatment, a divorce, a new relationship—and the philosophy, borrowed from an amateur mason he had once heard of, which he chose to use as a guideline and also in his story:

“I listened to the birdsong and then the bricks went awry, but don’t worry, we’ll make a new one.

Listen to the birdsong as long as you can.”

### Bridging the Past and the Future

**Figure 3.**
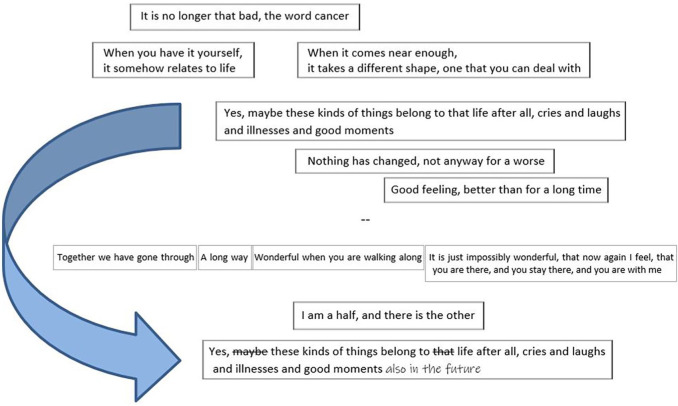
Bridging the past and the future, couple, the follow-up interview.

The ability of the participants to bridge the past and the future becomes manifest in the stories in several ways. Above all, it shows as a capability to include the future in one’s Storyboard, varying from a plan to celebrate the recovery, to plans to return to work, to travel, to renovate a house, even to build a new one, or to dream of a new kind of life and self. It also emerges through looking back at the illness experience and then forward to considerations of the future. Furthermore, it shows in the story continuations constructed with Clip(s) picked out from the original story indicating the evolution of meaning over time. Figure 3 presents an example of a story continuation of this type, simultaneously illustrating the expressiveness of the Clip Approach: a small change in one Clip and the new location in the sequence creates a dramatic change in meaning. The couple picked one Clip from the turning point of their story (the upper extract), modified it and moved it to the end of the story (the lower extract). The arrow indicates the move, the modifications are shown in strikethrough and Ink Free font. This example also illustrates the functioning of the Clips as a real-time instrument, for the purposes of processing one’s life story and a rupture experience.

### Embedding the Illness Experience to One’s Life Story

**Figure 4.**
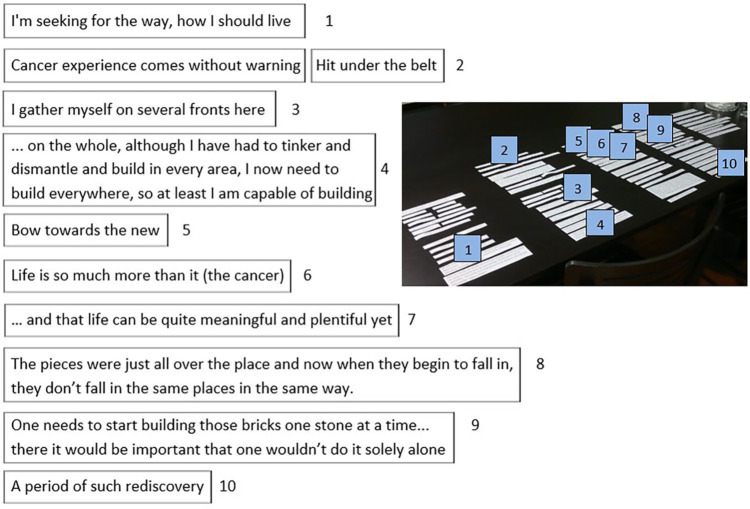
Sprinkling the cancer experience in the chronological continuum of the life story, patient, the fifth interview.

The Clip Approach enables embedding the illness experience into one’s life story in various ways. Figure 4 presents an example of sprinkling the illness experience in the chronological continuum as a thread intertwining within the whole life story.

The illness experience could also be embedded into the life story as an entity in a chronological continuum or through thematical parsing, or as a combination of these. The visual shapes of the stories provided space for creativity. They seemed to reflect the teller’s personal style, the content of the story, and even the relationship between the spouses. The stories in Figure 1 give four examples. First, the careful layout of the columnar life story having space above the first and below the last column, as if something would have been prior to (the past) and something would come after (the future)—

Somebody may say that there is enough of a story, when you tell what all you have achieved and done, you have a family . . . but maybe my life story is such that I have for a pretty long time sought myself

Then, the story in spacious rows allowing parallel Clips to exist on top of and under the rows, the core question of the story being the treatment choice—“*Tightrope walking*”. After that, the story in an unclassified form looks like the trunk of a tree growing from the left root (the individual history of the wife) above the right root (the individual history of the husband) as leaning on it—“*I guess we will not be given more burden than we can carry*”. And finally, the story as a relaxed pile, leaving the last Clip on top of the pile—“*The journey continues, life continues*”.

**Figure 5.**
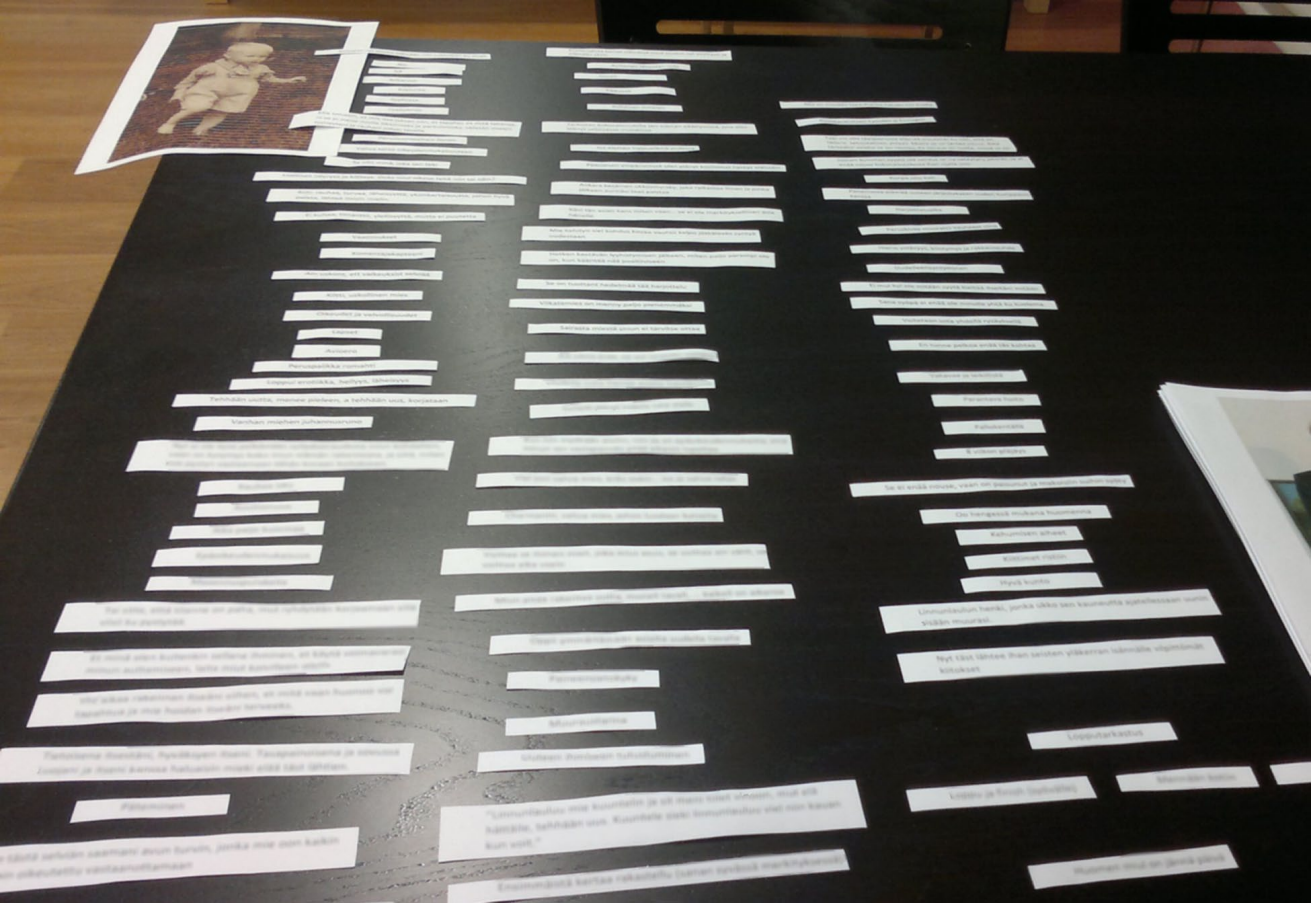
A story with additional visual elements, patient, the fifth interview.

With one participant, the Storyboard was supplemented with additional visual elements he had happened to bring to the meeting without knowing he would encounter other visual materials. When I asked him to create his story, he wondered: “*Why did I, for some strange reason, think in the same way? I brought two photos of me here, I thought (gives a laugh) I would talk a bit based on these to you.*” When his story was ready, I placed the photographs (one presenting him as a toddler, the other taken quite recently) where they seemed to belong. “*Between these two, it settles now, all these*” stated the man as this happened (Figure 5).

What comes to the two options for the length of the interview period, there was no difference in terms of the applicability of the Clip Approach. Both allowed for deep and subtle discussions, and conscious reflection, according to the participants, took place primarily in the interviews. Building one’s life story with the Clip Approach supported autobiographical reasoning, created continuity and enhanced the participants’ ability to prepare for the future:

Here, the whole life intertwines with the stories now on these clips, so this in a way sheds light on how one has lived and where one is now and how to get forward . . . I am now much more mature and able to face the life ahead again, I won’t worry about this cancer anymore (patient, the fifth interview)

Evaluating, interpreting, and making connections between different aspects of life and self could have been hard work. However, it contributed to the development of personality, identity, and personal growth, storytelling being at the heart of stability and change in self:

. . . what does a psychotherapist after all do more, this has functioned very well, and if I think, it is not easy sometimes, in fact, after an interview, I’ve felt, I’ve realized that one needs to use one’s brain a lot, one feels even a bit tired physically, but absolutely the fact that you have, you have put effort in doing these excerpts, it is, especially now, during the sixth time, I realize it even more clearly, that when we go through these, what I have said, and what I see . . . then all of a sudden it starts to build up, I see a bit what I think or how I change during this process . . . when one sees, that there has been a variety of mental development, and physical and mental hardship, and construction on different levels, and also development as a human, it brings forth stories like this (patient, the follow-up interview)

This five-columnar story is presented in Supplemental Attachment 3 in an anonymous, condensed form of 40 Clips (originally 87 Clips) as it flows.

## Discussion

This article introduces a new qualitative interview methodology developed to facilitate participants in the (re)creation of their life narratives after a rupture experience. The focus is on the use and the benefits of the visual artifacts (“the Clips”) that allow the interviewees to re-enter and re-consider their experience and life, and re-construct their narratives concerning them. The concepts of narrative and autobiographical reasoning as the research orientation proved useful in supporting the interviewees in coming to terms and embracing the challenge that cancer had brought into their lives. The accounts and the stories repeatedly showed that the core questions caused by the cancer dealt with the narrative identity, and handling these questions brought forth development in it. Hermeneutic elaboration—reflection and iteration — takes place throughout the new methodology, supporting the interviewees in working through the cancer experience and settling it in the context of the unfolding story of life. The resulting narratives are different from any other narrative data in the literature—both in how they were constructed, and in their final form. Hermeneutic philosophy is two-way interwoven in these “dense narratives” in which each Clip, as a detail of the narrative, stands for a larger whole known by the teller and the listener(s), and forms a new whole together with the other details of the story.

The Clips returned the oral accounts in a visual form allowing the illness experience and life to be perceived in a new way; they enabled and enhanced the process of accessing meaning and of generating new meaning (Reavey & Johnson, 2011). With the Clips, the participants could give shape and structure to their experience, and thus reach it from a nondiscursive side of producing oneself, strengthening their agency (Reavey & Johnson, 2011), and potentially arousing feelings of artistry, creativity, and productivity (Predeger, 1996). The Clip Approach brought forth new perspectives and options (Tomm, 1988), and facilitated in making connections between distant parts of life and self (McLean & Fournier, 2008) supporting continuity (Habermas & Köber, 2015), and bringing forth development in self (McAdams, 2013; Habermas, 2011) and personal growth (McLean & Fournier, 2008). In line with Martin’s (1998) method, it invited the participants to self-reflect on both the details and the whole, taking them a step beyond being confirmed as having been heard, therein allowing them to create and recreate themselves through narrative choices. For couples, the interviews were a window to the spouse’s thoughts, creating “other awareness” (Tomm, 1987b) and revealing new things even after decades of shared life. Building a story together enabled making sense as a common activity creating shared understanding, appreciation, and empowerment, the Clips working as tools for interaction and development of knowledge (Ewenstein & Whyte, 2007).

The Clips work in two ways analogously to psychotherapeutic work. They help the participants to develop self-observation, offering tools to organize it, thus helping to shift from an object position (as the victim of a life-threatening illness), to a subject position with personal agency (Leiman, 2012) and self-mastery (Kerr et al., 2020). The Clips also serve as testimonies in the same way as in narrative psychotherapy—being more than spoken word (Morgan, 2000). While usually associated with psychotherapy, in this study—under the influence of the realistic and imagined threats related to cancer—careful listening and intensive responding provides similar therapeutic tools for the participants enabling them, during the interview series, to be able to widely reach and utilize the narrative resources of both personal and social stock of stories (McAdams, 2013; Hänninen, 2004). In contrast to card sets used in some experiential techniques (Stein, 2007), the Clips directly refer to the participants’ own life experiences in their own words, being a confirmation of being heard (Martin, 1998) and strengthening participants’ dignity (Persson et al., 2020). Besides building on exact citations of the interviewees’ speech, the Clip Approach emphasizes the authority of the interviewees (Martin, 1998) through the possibility of them being able to select quotations, to add and remove content, to re-edit the story, and to continue it (Sparkes & Smith, 2008). The level of the use of existing Clips, and the paucity of changes to them, or writing new ones, advocates for the participants feeling the existing Clips relevant and adequate to their story. The stories constructed in the fifth interviews were also regarded as being valid after the follow-up period. Changes made rather illustrate development of meaning: the original building blocks of the Storyboard evolving to be extending building blocks for it.

Participants consistently report serial interviews as helpful (Murray et al., 2009), sometimes even more therapeutic than therapy interviews (Gale, 1992). Gale (1992) reported three reasons for this outcome. First, participants were acknowledged as sole owners of their experience, as holders of the knowledge, highlighted by the possibility to revisit and correct the information (video recordings of their therapy sessions) gathered earlier. Second, research interviews involved participants in a self-reflexive and recursive manner, enabling them to re-examine themselves, and thus gain flexibility and achieve multiple descriptions of their story. Third, the interviewer offered clarifying procedures (analogies) to help understand the situation of the participants, and to be enhanced by the participants leading to a collaborative re-telling of their story. All these dimensions can also be found in the Clip Approach, which acknowledges participants’ ownership of the knowledge, invites them to iterative self-reflection, and offers Clips as clarifying procedures to re-write their story as a collaborative effort. The participants found the interviews of this study as relieving, constructive, and therapeutic. During the cancer treatment appointments, they gave spontaneous feedback, how meaningful, supportive, and important the interviews were to them, subjectively even as important as the cancer treatment itself. Cancer patients have a great need to do something themselves (Melman & Newnham, 2011). For many, a cancer diagnosis is like a death sentence making one speechless and to feel hopeless, but it need not be. By actively and productively engaging with their experience, the participants could turn their uncertainty into possibilities, showing that meaning-making, enhanced agency, and coping are closely linked (Dauphin et al., 2020; Moran & Russo-Netzer, 2016). By telling and being listened to, a human being becomes aware, understands, and builds the world, oneself and one’s relationship with the surrounding world. Potential more favorable meanings for individual issues or events, or larger entities, at the widest for the whole life and/or for the self, strengthen, and increase well-being and mental health (Kerr et al., 2020). In this study, this psychological work was enabled on two levels, first on the level of the interviews, and then on the level of dense telling enabled by the Clips. The way the stories began to live and the experience to deepen indicate the benefits of the new method. The unfolding story of life could be widened and deepened by the Clip Approach.

### Evaluation

Owing to the fact that the interview methodology has not been widely applied, variation related to the interviewer has not yet been evaluated. On the other hand, there are also strengths in this study: (a) by involving men of different ages, the study gave a broad picture of a prostate cancer experience at various stages of life, and including spouses not only widened the perspective, but also enabled the participants to work through the cancer experience as a couple, creating shared agency, and strengthening mutual support; (b) sequential interviews over a longer timespan, together with careful listening and intensive responding built trust (Murray et al., 2009) and enabled deep personal exploration and working through the cancer experience from within the process; (c) transcribing the interviews without outsourcing the work to professionals supported immersing into the data serving both the interviewees and the research (McLeod & Balamoutsou, 2001); (d) the narrated was reflected back to the interviewees and meanings explored in a variety of ways on several levels, not only regarding the illness experience but also concerning the wider story of life; (e) the number of participants and interviews was high enough to show the applicability and advantages of the technique; and (f) the method allowed the stories to emerge, evolve, and continue (Sparkes & Smith, 2008).

Getting a serious illness, prostate cancer, and the cancer experience during the treatments, from the viewpoint of the patient and the spouse, were the framework of this study. The study does not, however, aim at providing knowledge generalized to this population, instead to the phenomenon of the added value of the use of visual artifacts as part of a psychosocial interview intervention in the context of a serious health crisis. One Clip presented in this report included personal data and was made generic. No other challenges to the ethical principles occurred.

### Implications

The resulting sequential unfolding of the narrative, the Storyboard, offers a new type of platform for the participants to reflect and negotiate their experience, and a new type of source of data for researchers to analyze (with for example visual, narrative, hermeneutic, discursive, or even poetic methods). Future research will be able to use the new methodology to investigate the working out of autobiographical ruptures caused by, for example, various illnesses, unemployment, divorce, retirement, or developmental changes such as transition to parenthood.

The methodology has not been tested by practitioners, but it is likely to offer tools for, for example, counselors, psychotherapists, medical doctors, nurses, and social workers. As such, the Clip Approach could be beneficial for environments where there is sufficient time for collaborative work, like psychotherapies. If the work intensity of the methodology can be reduced, for example, by extracting quotes directly from a recording, or using speech recognition technique for the transcription work, the Clip Approach offers encouraging development potential for a low-threshold psychosocial support method in various applicability areas. Potential target groups could include patients with a severe long-term illness in somatic treatment, clients in challenging life change situations in social work, and elderly people in the need of support for their life stories in care. The methodology suits different kinds of people, both clients and providers, if only they have enough of time, willingness and the ability to deeply interact in a series of respectful discussions. Paraphrasing Martin (1998), to speak is one thing, to hear is another, to confirm as having heard is yet another. To let one create and recreate oneself.

## Supplemental Material

sj-pdf-1-qhr-10.1177_1049732320982945 – Supplemental material for The Clip Approach: A Visual Methodology to Support the (Re)Construction of Life Narratives Click here for additional data file. Supplemental material, sj-pdf-1-qhr-10.1177_1049732320982945 for The Clip Approach: A Visual Methodology to Support the (Re)Construction of Life Narratives by Riikka Talsi, Aarno Laitila, Timo Joensuu and Esa Saarinen in Qualitative Health Research

sj-pdf-2-qhr-10.1177_1049732320982945 – Supplemental material for The Clip Approach: A Visual Methodology to Support the (Re)Construction of Life Narratives Click here for additional data file. Supplemental material, sj-pdf-2-qhr-10.1177_1049732320982945 for The Clip Approach: A Visual Methodology to Support the (Re)Construction of Life Narratives by Riikka Talsi, Aarno Laitila, Timo Joensuu and Esa Saarinen in Qualitative Health Research

sj-pdf-3-qhr-10.1177_1049732320982945 – Supplemental material for The Clip Approach: A Visual Methodology to Support the (Re)Construction of Life Narratives Click here for additional data file. Supplemental material, sj-pdf-3-qhr-10.1177_1049732320982945 for The Clip Approach: A Visual Methodology to Support the (Re)Construction of Life Narratives by Riikka Talsi, Aarno Laitila, Timo Joensuu and Esa Saarinen in Qualitative Health Research
