# Supplementary material for: The Clip Approach: A Visual Methodology to Support the (Re)Construction of Life Narratives
Source: Qual Health Res. 2021 Feb 11;31(4):789–803. doi: 10.1177/1049732320982945 (PMC7885092; doi:10.1177/1049732320982945)
Supplement: sj-pdf-1-qhr-10.1177_1049732320982945 – Supplemental material for The Clip Approach: A Visual Methodology to Support the (Re)Construction of Life Narratives [file sj-pdf-1-qhr-10.1177_1049732320982945.pdf]

Attachment 1. Interview themes

Cancer treatment

- Phase
- Treatments
- Needs in different phases
- Knowledge
- Care personnel

Physical well-being

- Physical effects of cancer and treatments and needs arising from them
- Pain
- Fatigue
- Sleep
- Appetite
- Nausea
- Urination
- Defecation
- Sexual effects

Mental well-being

- Mental effects of cancer and treatments and needs arising from them
- Emotions
- Tension
- Fear
- Worry
- Grief
- Death
- Acceptance
- Adaptation
- Hope
- Manhood
- Identity
- Attitude
- Thinking

Social well-being

- Social effects of cancer and treatments and needs arising from them
- Need for social support
- Meaning of social support
- Human relationships
- Friends
- Help and support

Patient and spouse

- The effects of cancer and treatments on the relationship between the patient and the spouse and needs arising from them
- Relationship between patient and spouse
- Encountering difficult emotions related to the illness
- Effects of cancer on sexuality and intimate life
- Learning to live with changes in the relationship
- Closeness, openness, compassion and neediness
- New normal
- Interaction
- Meanings and shared meanings
- Supporting the spouse
- Well-being of both spouses
- Ability to encounter cancer together
- Commitment
- Common story

Family

- The effects of cancer and treatments on family members and needs arising from them
- Support needed by family members
- Help and support given by family members

Work and leisure

- The effects of cancer and treatments on work and leisure and needs arising from them
- Work
- Leisure
- Action

Future

- New perspectives
- New direction
- Possibilities
- In spite of the illness
- Experiencing one’s health
- Rehabilitation
- Healing
- Well-being
- Quality of life

Cancer experience and life story

- The whole
- Wonder
- Openness to change
- The element of surprise
- Humor
- Mystery
- Trust
- Own voice
- Meaningfulness
- Reasonableness
- Coping
- Achievements
- Strengths
- Priorities
- Values
- Gratitude
- Mental growth
