# Supplementary material for: The Clip Approach: A Visual Methodology to Support the (Re)Construction of Life Narratives
Source: Qual Health Res. 2021 Feb 11;31(4):789–803. doi: 10.1177/1049732320982945 (PMC7885092; doi:10.1177/1049732320982945)
Supplement: sj-pdf-2-qhr-10.1177_1049732320982945 – Supplemental material for The Clip Approach: A Visual Methodology to Support the (Re)Construction of Life Narratives [file sj-pdf-2-qhr-10.1177_1049732320982945.pdf]

Attachment 2. Cancer experience as a composition of short extracts of several Storyboards of several participants, the 5th interviews, hand-written text in Ink Free font

A thump

My visions made a front flip

My thoughts hit the idle

Is the man beginning to unravel?

--

Radiotherapy | Tightrope walking | Radical operation

--

This isn't an easy length

You have been in your thoughts | A couple of nights went quite poorly

Running out of all strength | You cried, I comforted, in another situation the other way around

A horrible fear of losing

--

Spring, light, warmth

We sat on a cliff and there was a pair of goldeneyes in spring cleaning

To be close to each other

--

I can hear, what you have thought | Stronger than I believed | A cat finds its claws when needs to climb a tree

--

I believe that I will recover

--

It is lighter to go

It has happened during a short period

I will still go far

I have been lucky

Death does not frighten me anymore

--

It went like it was supposed to
