# Supplementary material for: The Clip Approach: A Visual Methodology to Support the (Re)Construction of Life Narratives
Source: Qual Health Res. 2021 Feb 11;31(4):789–803. doi: 10.1177/1049732320982945 (PMC7885092; doi:10.1177/1049732320982945)
Supplement: sj-pdf-3-qhr-10.1177_1049732320982945 – Supplemental material for The Clip Approach: A Visual Methodology to Support the (Re)Construction of Life Narratives [file sj-pdf-3-qhr-10.1177_1049732320982945.pdf]

Attachment 3. The story of a participant, the 5th interview

I

A distant father and an over-protective mother are that kind of a combination, which sets the children forward, to achieve things

A nascent relationship *A divorce process*

Daughters

I'm seeking for the way, how I should live

If now something got broken... maybe the bike broke down or one must take a little break... so they are then perhaps the kind of things that can be built or repaired

Somebody may say that there is enough of a story, when you tell what all you have achieved and done, you have a family... but maybe my life story is such that I have for a pretty long time sought myself

II

Cancer experience comes without warning *Hit under the belt*

You are wondering whether you will die or not *It makes the finiteness of life concrete*

I hadn't planned for this kind of a halt

It's as if you were bike-riding... and a truck hits you and you fly to a ditch... you pick yourself up, are all the bones intact, cough up sand and dust, and wipe bloody wounds.

I get started, when there is a crisis *I make pretty big decisions quickly*

A longer break *I gather myself on several fronts here*

The cancer diagnosis stopped the nascent stepping forward, now I am again gathering fragments

It stopped me and made me think things over, and that thinking, and processing of things has been in fact a surprisingly big thing

Nothing really irreversible maybe, however, has occurred

... on the whole, although I have had to tinker and dismantle and build in every area, I now need to build everywhere, so at least I am capable of building

III

Fortunately, I acted

Bow towards the new *Bow to that direction, where you are going*

It can be painful, but there can… be then quite good consequences as well

Life is so much more than it (the cancer)

... and that life can be quite meaningful and plentiful yet

It could have taken all the interest or the possibility to that kind of a relationship, which would have been a big loss

Plus, it could have taken away the desire to live or the ability to rebuild those bricks again

In a battle mode, I began to breathe again, started to look at the bigger picture, how this has influenced my view of life, where I want to move on, to which tribe to settle

Clarity, that I wouldn’t get entangled in wires

Life fits into that beauty box

IV

The pieces were just all over the place and now when they begin to fall in, they don’t fall in the same places in the same way.

One needs to start building those bricks one stone at a time... there it would be important that one wouldn’t do it solely alone

I would like to get out of that kind of self-centeredness, that I, I, I, I achieve

It's also important to clean up one’s tracks, to make things so that one can call it even with one’s past

A period of such rediscovery

V

It has taken quite a long time for me to begin to think that the context, or crowd with whom you do things, has a lot of significance

Own life or tribe is building up

Together with others one can achieve much more, if the values and goals are good

True happiness is when you immerse yourself in something important, interesting, demanding
